# Investigation of anti-diabetic potential and molecular simulation studies of dihydropyrimidinone derivatives

**DOI:** 10.3389/fendo.2022.1022623

**Published:** 2022-10-12

**Authors:** Umair Ilyas, Bisma Nazir, Reem Altaf, Syed Aun Muhammad, Hajra Zafar, Ana Cláudia Paiva-Santos, Muhammad Abbas, Yongtao Duan

**Affiliations:** ^1^ Henan Provincial Key Laboratory of Children’s Genetics and Metabolic Diseases, Children’s Hospital Affiliated to Zhengzhou University, Zhengzhou University, Zhengzhou, China; ^2^ Riphah Institute of Pharmaceutical Sciences, Riphah International University, Islamabad, Pakistan; ^3^ Department of Pharmacy, Iqra University Islamabad Campus, Islamabad, Pakistan; ^4^ Institute of Molecular Biology and Biotechnology, Bahauddin Zakariya University, Multan, Pakistan; ^5^ School of Pharmacy, Shanghai Jiao Tong University, Shanghai, China; ^6^ Department of Pharmaceutical Technology, Faculty of Pharmacy, University of Coimbra, Coimbra, Portugal; ^7^ REQUIMTE/LAQV, Group of Pharmaceutical Technology, Faculty of Pharmacy, University of Coimbra, Coimbra, Portugal

**Keywords:** Dihydropyrimidinones, alpha-glucosidase, molecular docking, anti-diabetic, *in-silico*

## Abstract

In an attempt to find new targets for α-amylase and α-glucosidase for the treatment of type 2 diabetes mellitus, the present study aims in determining the anti-diabetic potential of synthesized dihydropyrimidinone derivatives. The *in vitro* α-glucosidase and α-amylase inhibitory activity was performed and the molecular docking analysis of the ligand in the active binding site of target protein was determined. The results revealed significant percent inhibition of α-glucosidase by the compound *6-benzyl-4-(4-hydroxyphenyl)-3,4,6,7-tetrahydro-1H-pyrrolo[3,4-d]pyrimidine-2,5-dione* (compound A). The active compound showed 81.99% inhibition when compared to standard ascorbic acid having percent inhibition 81.18%. The IC_50_ of active compound (A) showed to be 1.02 µg/ml. The molecular docking analysis revealed that the ligand bound to the active binding site of protein with the lowest binding energy of -7.9 kcal/mol that was also significantly similar to standard having -7.8 kcal/mol binding energy. The molecular dynamic simulation studies also revealed stable binding of ligand in the active binding site of protein with low RMSD of 1.7 Å similar to the protein RMSD 1.6Å In conclusion, the study revealed a potential new target against α-glucosidase to treat type 2 diabetes mellitus.

## Introduction

Incidence of diabetes mellitus have increased globally. Around the world, diabetes mellitus is a major health issue associated with decreased quality of life, increased morbidity and mortality and increased burden of health care costs. Current diabetic therapies aimed at lowering blood sugar levels but did not address the root cause of diabetes mellitus as progression of insulin deficiency was not improved. Oral drugs for diabetes mellitus have many problems associated with treatment including loss of beta cell function and cause increase in impaired insulin secretion. The pathophysiology of this disease was not completely understood. Aside from that, current anti-diabetic drugs are not improving the side effects associated with anti-diabetic drugs including cardiovascular risk (1).

Traditionally, different treatment options are available which include a variety of oral hypoglycemic agents but none of which effectively control glucose levels indefinitely. However, although combination therapies are available but they have limitations in their function to end stage in type 2 diabetes mellitus (2). Several new drugs have become available in some countries nowadays, but higher cost of these drugs along with absence of clinical safety need to be addressed by health care organizations and clinicians (3). We have to consider the selection of newer anti-diabetic drugs but due to inconsistent reporting, we are not able to analyze the effect of each new anti-diabetic drug (4). Managing complications with diabetes mellitus has always been a challenge for physicians (5). To address or delay the complications associated with treatment of diabetes mellitus, there is a need to adopt a systematic approach for managing diabetes mellitus including self-management training (6, 7). Government needs to develop long term policies to deal with the growing burden of diabetes mellitus (8).

Dihydropyrimidinones (DHPM) are heterocyclic compounds with a pyrimidine moiety in the ring core and has been of interest in medicinal chemistry for decades due to its versatile bioactivity (9). Dihydropyrimidinones play important role in synthesis of RNA and DNA (10). They have great number of activities including anti-diabetic, anti-inflammatory, anti-hypertensive, anti-cancer, and anti-malarial and many more. Pyrimidine nucleus is the main reason of many therapeutic applications. Substitution in pyrimidine moiety offers an opportunity for development of newer drugs with better efficacy (11). Interestingly, the antidiabetic potential of dihydropyrimidinones is still not fully explored which give us the opportunity to determine the role of dihydropyrimidinones as anti-diabetic agents (12, 13). In type 2 diabetes mellitus patients, alpha glucosidase and alpha amylase enzymes perform short term effect and decreased blood glucose level. These enzymes helps to retard glucose absorption to decrease postprandial hyperglycemia (PPHG). Inhibition of these enzymes helps to reduce the rate of digestion of carbohydrates so less amount of glucose is absorbed because the carbohydrates are not broken down into glucose molecules. As both these enzymes helped to reduce the risk of developing diabetes mellitus (14).

In the present study, the antidiabetic potential of dihydropyrimidinone derivatives were explored. These derivatives were synthesized in a previous study and anti-cancer potential of these compounds were studied. The study showed promising anti-cancer activity against breast cancer cell line MCF7 (15). Moreover, the alkaline phosphatase activities of these compounds were also studied that showed potential of these compounds as alkaline phosphatase inhibitors (16). Keeping in view, of these potential activities of synthesized derivatives of dihydropyrimidinones the anti-diabetic potential of *6-benzyl-4-(4-hydroxyphenyl)-3,4,6,7-tetrahydro-1H-pyrrolo[3,4-d]pyrimidine-2,5-dione* (compound A), 4*-(4-hydroxyphenyl)-6-octyl-3,4,6,7-tetrahydro-1H-pyrrolo[3,4-d]pyrimidine-2,5-dione, 6-(4-chlorobenzyl)-4-(4-hydroxyphenyl)-3,4,6,7-tetrahydro-1H-pyrrolo[3,4-d]pyrimidine-2,5-dione* (compound **C**) and *6-(4-fluorobenzyl)-4-(4-hydroxyphenyl)-3,4,6,7-tetrahydro-1H-pyrrolo[3,4-d]pyrimidine-2,5-dione* (compound **D**) were thought to be studied. The alpha amylase and alpha glucosidase activities will be performed to evaluate the anti-diabetic activity. Moreover, the molecular docking studies and the molecular simulation studies of these compounds will be performed to analyze the binding energy and the protein changes occurring during protein ligand interaction.

## Methodology

### α-amylase inhibition assay

In α**-**amylase inhibition assay, the reaction mixture containing 15 μl phosphate buffer (pH 6.8), 25 μl α-amylase enzyme (0.14 U/ml), 10 μl sample (1 mg/ml DMSO) and 40 μl (2 mg/ml in potassium phosphate buffer) starch solution was incubated at 50°C for 30 min in 96 well plate. Then 20 μl of 1 M HCl was added to stop reaction. Afterwards 90 μl of iodine reagent (5 mM iodine, 5 mM potassium iodide in phosphate buffer) was added to each well. Blank was prepared by adding dimethyl sulphoxide and the phosphate buffer instead of sample and enzyme solution respectively, whereas negative control was prepared by adding dimethyl sulphoxide in place of sample. Ascorbic acid (250 μM) was used as a positive control. Absorbance of the reaction mixture was measured at 540 nm. The activity was expressed as the percent α-amylase inhibition and calculated by the following equation:


%α-amylase inhibation = (Os-On)/(Ob-On) × 100

Where, On = Absorbance of negative control, Os = Absorbance of sample and Ob = Absorbance of blank well.

Samples which depicted 50% inhibition in the initial screening were further analyzed at lower concentration to find IC_50_ (17).

### Selective alpha glucosidase inhibition assay

An aliquot of 25 μl of substrate solution was mixed with the 69 μl of phosphate buffer saline and 5 μl of test sample. Enzyme solution 1 μl was then added to respective wells of 96 well plate. Zero hour reading was recorded at 405 nm using microplate reader followed by an incubation period of 40 minutes at temperature of 37°C. Afterwards, 100 μl of sodium bicarbonate solution was added to the above solution to stop the reaction. Absorbance was noted at 405 nm. Procedure was repeated for preparation of negative and the positive control containing dimethylsuphoxide and ascorbic acid respectively.


% inhibition = (Ac-As/Ac) × 100

Ac and As = Absorbance of control and absorbance of sample respectively.

Samples which depicted 50% inhibition in initial screening were further analyzed at lower concentration to find IC_50_ (17).

### Molecular docking

#### Ligand selection and optimization

The synthesized chemical structures A, B, C and **D** were used as ligand molecules and their structures were drawn using chemsketch software. The files were saved in.mol format and converted to.pdb format using Discovery Studio 4.0. The ligands were then prepared for docking adapt using Autodock Tools 1.5.6 and converted to.pdbqt format. The AutoDock Tools prepared the ligands by adding polar hydrogens, gasteiger charges were added to all atoms, and non-polar hydrogens were removed from all the ligands.

#### Accession of target protein

The protein structures of alpha glucosidase (18) was selected for the protein ligand binding analysis. The protein was obtained from protein data bank having PDB ID: 5KZW (www.pdb.com).

#### Target protein optimization

For protein optimization the protein was prepared using mgl tools of AutoDock. The ligands and water were removed from the crystallized protein structure using Discovery studio 4.0. The protein was prepared using AutoDock Tools and saved in.pdbqt format.

### Validation of docking

The validation of docking procedure was performed by redocking the obtained pose on the same active binding site of target protein. The grid parameters were kept unchanged and all the protocols were kept constant. The binding of ligand on the active binding site with less deviation when compared to actual complex validates the docking protocol. The redock complex was superimposed using the Discovery studio 4.0 and PyMol 2.3 on the reference complex. The redock complex of active ligand as well as the standard acarbose was superimposed and the root mean sqaure deviation was calculated.

### Molecular dynamic simulation studies

The use of molecular dynamic simulation tools have helped us in many ways in the determination of structural stability of a protein as well as the protein-ligand complexes under physiological conditions. The most active ligand showing significant anti-diabetic activity was used for MD simulation studies. In molecular docking studies a static view of ligand is provided in the active binding site of protein thereby, predicting the ligand period binding status, whereas, in molecular dynamic simulation, the movement of atoms is calculated using the Newton’s classical equation of motion over a specific period of time. The Desmond (2012) module of Schrodinger software was used to perform the simulation studies. The simulations were run over a 100 nanosecond trajectory period using the OPLS-2005 force field. The physiological environment was simulated to predict the ligand protein binding status and the preprocessing of ligand-protein complex was done using the protein Preparation Wizard of Maestro. The complexes were optimized and minimized as well. In an orthrombic box, the complex was predefines in a TIP3P water model. The Na and CL ions were added to neutralize the overall charge of the system. The pressure of 1.0132 bar and temperature of 300K was kept constant while keeping the box volume minimized. The root mean swaure deviation (RMSD) was used to evaluate the stability of simulation studies for all the trajectories^5c, 11 5c, 11^.

## Results

### α-amylase inhibition assay

The tested compounds were subjected to examine the inhibitory potential against α-amylase. All of the compounds showed less than 50% inhibition having no potential activity against α-amylase.

### Selective alpha glucosidase inhibition assay

Four test compounds were investigated for their antidiabetic activity through alpha glucosidase inhibition assay. While only one compound (*A*) showed antidiabetic activity against standard ascorbic acid. The compound *A* which depicted 50% inhibition in initial screening were further analyzed at lower concentration to find IC_50_. **Table 1** showed results of alpha glucosidase inhibition assay.

**Table 1:** Percentage inhibition and IC_50_ of tested compounds using α-glucosidase inhibition assay.

Sr.No	Sample name	% Inhibition	IC_50_ (µg/ml)
1	**A** compound	81.99	1.02
2	**B** compound	31.27	NA
3	**C** compound	34.14	NA
4	**D** compound	32.35	NA
5	Negative control (DMSO)	–	NA
6	Positive control (Ascorbic acid)	81.18%	NA
7	Blank	–	NA

Sample concentration = 25μg/ml; Positive control concentration = 25μg/ml

NA, not applicable. Bold values indicate the compound A, B, C, and D.

### Molecular docking

**Figure 1:**
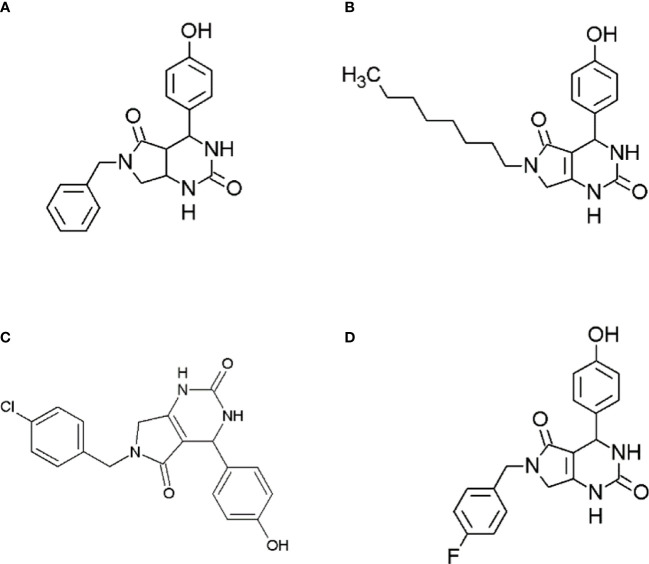
Structures of synthesized dihydropyrimidinone derivatives, **(A)** 6-benzyl-4-(4-hydroxyphenyl)-3,4,6,7-tetrahydro-1H-pyrrolo[3,4-d]pyrimidine-2,5-dione (compound A); **(B)** 4-(4-hydroxyphenyl)-6-octyl-3,4,6,7-tetrahydro-1H-pyrrolo[3,4-d]pyrimidine-2,5-dione (Compound B); **(C)** 6-(4-chlorobenzyl)-4-(4-hydroxyphenyl)-3,4,6,7-tetrahydro-1H-pyrrolo[3,4-d]pyrimidine-2,5-dione (compound C); and **(D)** 6-(4-fluorobenzyl)-4-(4-hydroxyphenyl)-3,4,6,7-tetrahydro-1H-pyrrolo[3,4-d]pyrimidine-2,5-dione (compound D).

**Figure 2:**
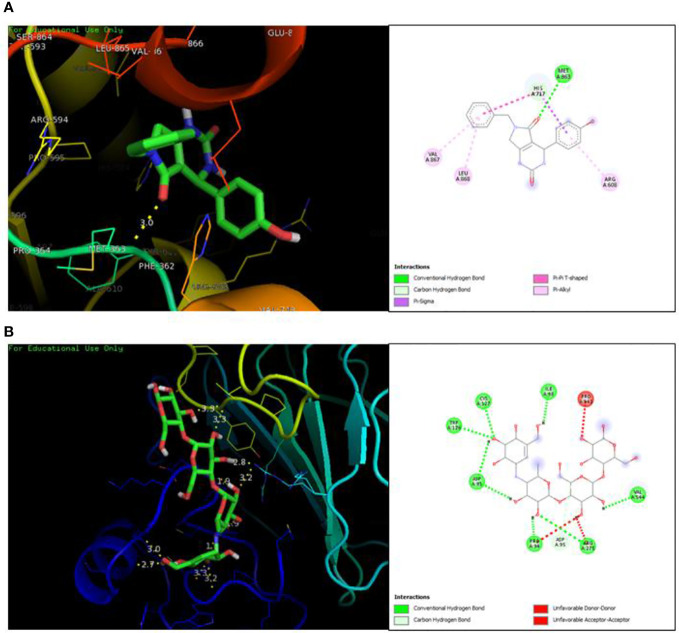
Protein-ligand interaction showing compound A **(A)** and standard acarbose **(B)** in the active binding site of protein α-glucosidase.

The docking analysis revealed efficient binding of ligand within the active binding site of protein having lowest binding energy of -7.9 kcal/mol as compared to standard acarbose showing lowest binding energy of -7.8 kcal/mol **Figure 1**. **Table 2** shows the binding scores of active ligand and standard ascorbic acid. The interaction analysis revealed that the active ligand formed one stable conventional hydrogen bond with MET363. Some pi-alkyl and pi-pi stacked interactions were also observed with VAL867, LEU868 and ARG608. A pi-sigma interaction was observed between phenyl ring and HIS717 (**Figure 2A**). The amino acid interaction of standard acarbose was also visualized and the results revealed nine conventional hydrogen bonding with amino acid residues CYS127, TRP126, ASP91, VAL544, PRO94, ARG275 (**Figure 2B**).

**Table 2:** Calculations of binding affinity values of ligand.

Ligand	Binding Affinity(Kcal/mol)	RMSD/UB	RMSD/LB
Compound ***A***	-7.9	0	0
Compound ***A***	-7.9	45.222	42.568
Compound ***A***	-7.8	6.098	2.512
Compound ***A***	-7.6	3.648	2.234
Compound ***A***	-7.6	33.33	30.397
Compound ***A***	-7.6	5.423	3.36
Compound ***A***	-7.5	44.849	42.431
Compound ***A***	-7.3	42.034	39.038
Compound ***A***	-7.3	29.39	26.432
Acarbose	-7.8	0	0
Acarbose	-7.7	38.975	37.361
Acarbose	-7.4	13.659	11.103
Acarbose	-7.4	12.406	10.744
Acarbose	-7.4	29.074	28.218
Acarbose	-7.2	26.534	25.172
Acarbose	-7.1	14.279	11.451
Acarbose	-7.1	29.109	28.068
Acarbose	-7.1	13.77	11.844

Bold values indicate the compound A, B, C, and D.

### Validation of docking

**Figure 3:**
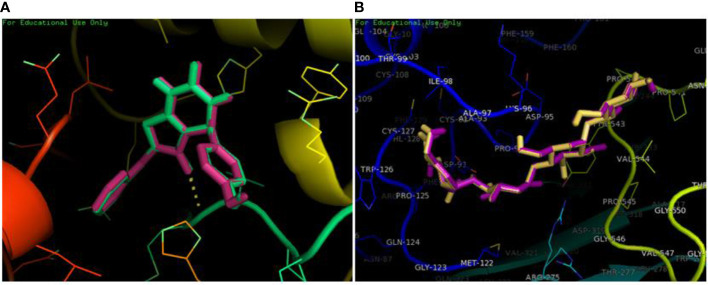
Superimposition of redocked complex with the reference complex. **(A)** The redocked complex of active compound is superimposed onto the reference conformation in the active binding site of protein. **(B)** The redocked complex of standard acarbose is superimposed onto the reference conformation in the active binding site of protein.

The redocking was done by docking the best pose in the active binding site of target protein in order to validate the docking protocols and to ensure the docking efficiencies. The binding of ligand was observed in the same active site as that of reference after redocking having rmsd of 0.1 kcal/mol. The amino acid involved in interactions were MET363 showing bonding with the oxygen atom and one carbon hydrogen bond with HIS717. Similar pattern of pi-pi T shaped and Pi-alkyl interactions were observed with amino acid residues ARG608, VAL867 and LEU868. PyMOL was used to superimpose the native co-crystallized ligand in the docked complex. A very low rmsd of 0.1 kcal/mol suggested the validation of docking procedure (**Figure 3A**).The forest green complex shows the reference conformation attained during docking while the pink complex signifies the redock complex of active compound in the active binding site of a-glucosidase. Similarly, the yellow complex shows the redocked complex of acarbose in the active binding site of a-glucosidase and pink complex represents the reference complex attained during docking having rmsd of 0.1 kcal/mol (**Figure 3B**).

### Molecular dynamic simulation studies

#### RMSD analysis

**Figure 4:**
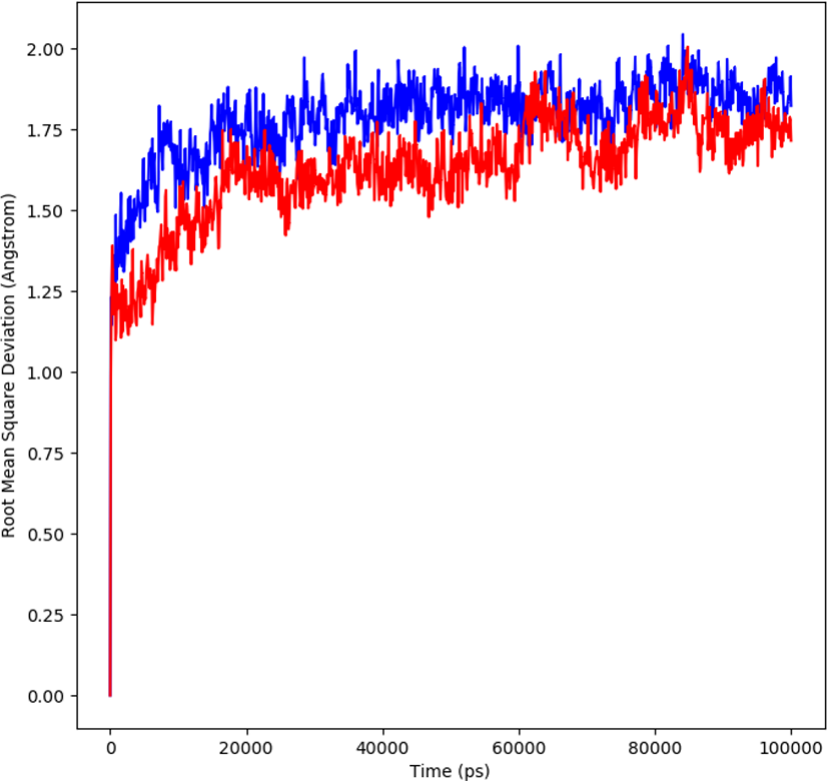
RMSD profile of protein-ligand complex (red) and protein complex (blue) for 100 ns of MD simulation.

The molecular dynamic simulation studies were carried out at 100 ns and the changes in the protein ligand complex was determined using the root mean square deviation (RMSD). RMSD determines the flexibility of the protein, the equilibration and the average distance between the atoms of the proteins. The plot generated depicts the RMSD showing the complexes reached the stable form. For 100 ns trajectory, the complex showed stability throughout the simulation. The average RMSD for α-glucosidase-compound A complex was 1.7Å, a slight fluctuation was observed at 10-20ns, the equilibrium was then achieved after 40ns. The complex remained stable afterwards. The average RMSD for protein complex was 1.6Å, a similar pattern in fluctuation was observed for this complex over a 10 -20 ns. The equilibrium was attained after 40 ns which remained stable throughout simulation. The system stability is determined by a low RMSD values followed by little fluctuations (19). During the simulation trajectories, the results suggested stable interaction and acceptable range for the studied complex (**Figure 4**).

#### RMSF analysis

**Figure 5:**
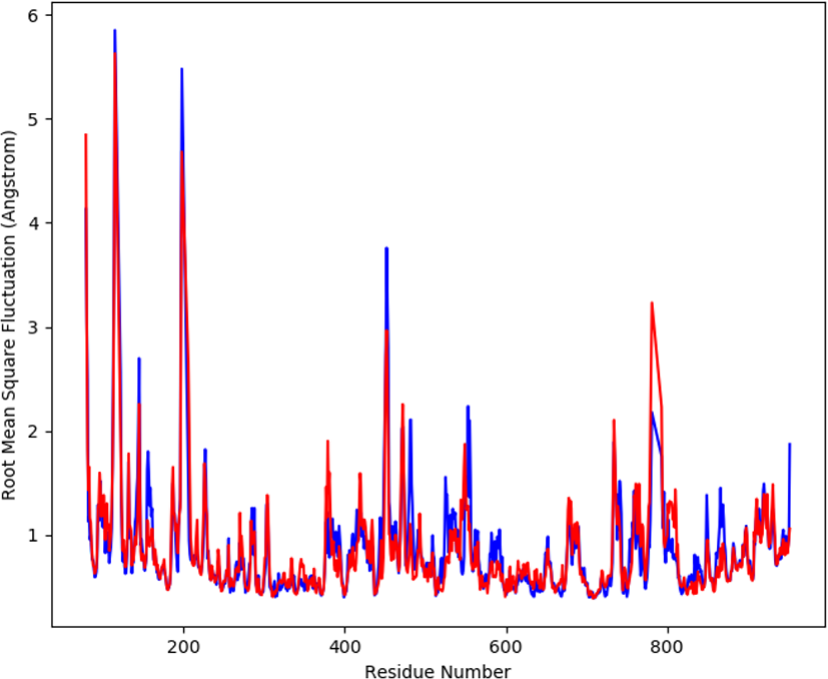
The graph displaying the RMSF values of Cα atoms for 100 ns trajectories.

The average movement of atoms was measured by root mean square fluctuation (RMSF) at a specified temperature and pressure. The flexible region of the protein is determined by RMSF, moreover, the regions of structures that shows fluctuation to overall structure of protein is also evaluated by RMSF. A low value of RMSF is suggestive of a good stable system, a high RMSF is predictive of greater flexibility of the system during the simulation trajectories. At 100 ns period, the plot of RMSF was generated to observe the fluctuation in the residues of the protein a-glucosidase and the complex. The **Figure 5** shows the flexibility of residues in the protein and complex as well as the fluctuation in the residues. The plot shows less fluctuation and good stability for both the complexes.

#### Radius of gyration

**Figure 6:**
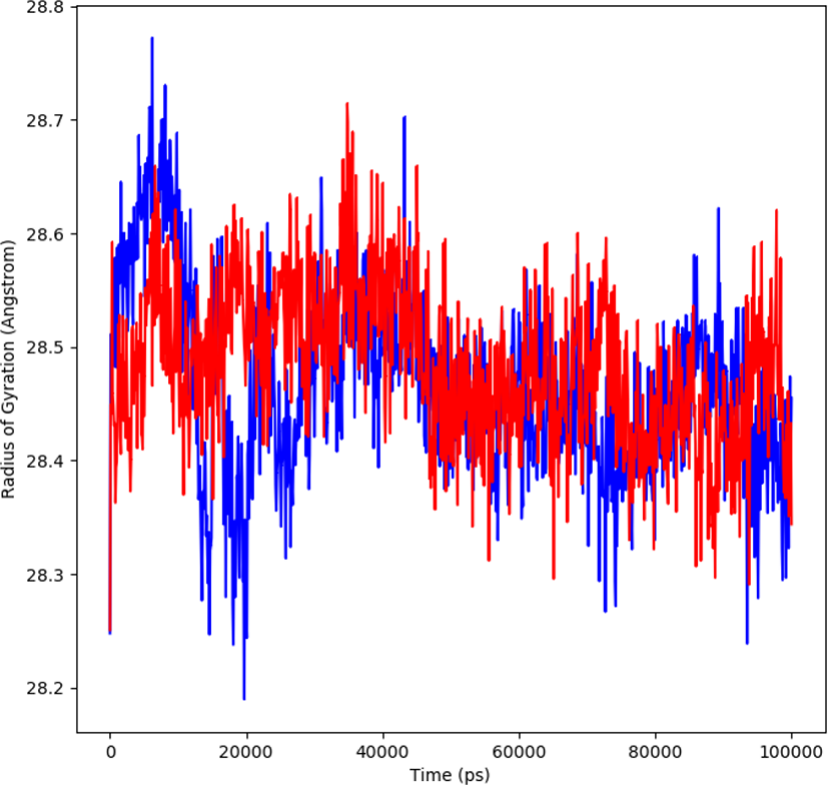
Rg plot representing changes observed in the conformational behavior of protein-ligand complexes (red depicting protein-ligand complex, while blue indicating protein) for 100 ns MD trajectories.

The changes in compactness between the ligand and protein complex is assessed by the radius of gyration (Rg). The stable folding and unfolding of protein and its complexes is also determined by Rg. A high Rg suggest a low compactness of protein-ligand complex. The average Rg of ligand showed to be 28.6 Å ranging from 28.2 to 28.6 Å (red). The protein complex average Rg was 28.4 Å ranging from 28.2-28.7Å (blue) (**Figure 6**). During MD simulation, a stable protein folding is determined by maintenance of a relative steady value of Rg, if Rg changes over time it indicates the unfolding of the protein (20, 21). The consistent values of Rg were observed form the results and a good stability was observed due to similar behavior of compactness by the complex.

#### Solvent accessible surface area

**Figure 7:**
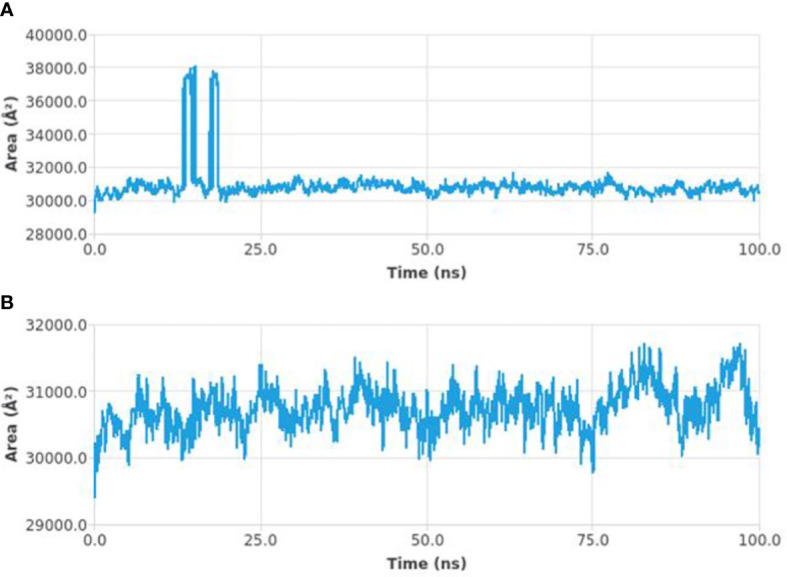
SASA curves highlighting the changes in the solvent accessibility of the studied protein complexes (protein-ligand complex **(A)** and protein complex **(B)**) during 100 ns MD trajectories.

The solvent accessible surface area (SASA) measures the interactions between the complexes and the solvents and predicts the conformational changes taking place during interaction. Therefore, the SASA of the protein-ligand complex was determined. **Figure 7** depicts the SASA plot verses the time for both the protein-ligand complex and the protein complex. The average SASA value for protein-ligand complex was 30906.17 nm2 (red), similarly, the average SASA value for protein complex was 30736.86 nm2 (blue). The values suggested no significant changes on the protein structure and the values were found to be relatively stable over the period of 100 ns. The calculations showed that the complex had a significant similar value of SASA with that of reference protein (22).

#### Interaction energy

**Figure 8:**
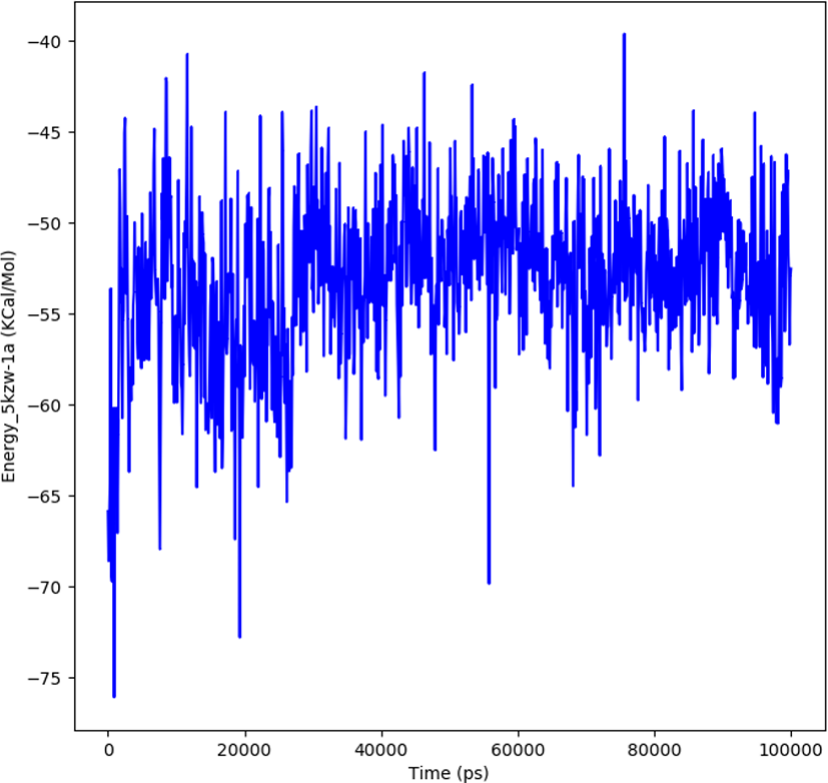
The plot illustrating the interaction in the form of free energies of binding between protein-ligand complex.

The interaction energy is calculated to determine the strength with which protein and ligand binds with each other. The free energies of interaction between the ligand and protein structure is calculated by a detailed analysis to validate the binding energies generated by the molecular docking analysis. The interaction energy for the protein-ligand complex at 100 ns was found to be -53.16 kcal/mol. The highest energy attained was -69.3 kcal/mol and showed favorable binding of ligands with hTERT. The results validated the molecular docking results and showed the potential of ligand as a-glucosidase inhibitor. **Figure 8** represents the interaction energy attained by the protein-ligand complex over a period of 100 ns.

#### Hydrogen bond analysis

**Figure 9:**
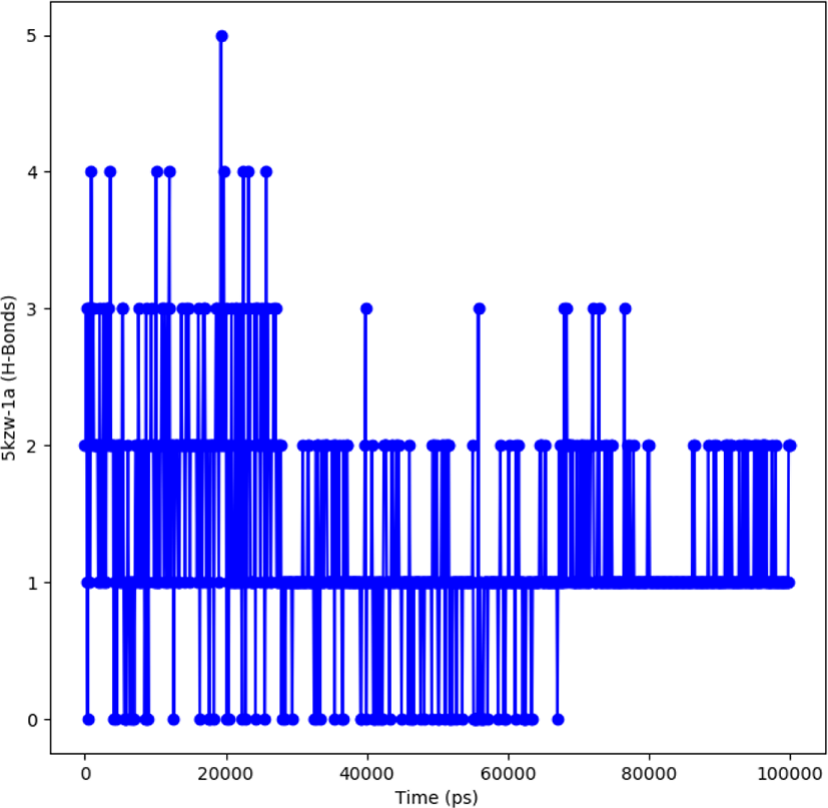
Representation of hydrogen bonding pattern observed for protein ligand complexes (adapalene-red and tamsulosin-blue) for 100 ns MD simulation.

The protein-ligand complex is considered stable depending on the hydrogen bonding between the ligand and the receptor. The hydrogen bonding also determines the drug specificity, metabolism and adsorption. The simulation results showed that the complex formed four hydrogen bonds concluding effective and tight bounding of ligands in the active biding site of α-glucosidase protein. The total number of hydrogen bonds formed in the complex during the 100 ns simulation period is shown in **Figure 9**.

## Discussion

Diabetes mellitus is a progressive metabolic disease characterized by in ability of the body to control blood sugar levels resulting in chronic hyperglycemia (23). Several traditional medications for the treatment of type 2 diabetes increases the insulin action and its secretion for β-pancreatic cells (24). Some of the digestive enzymes present in the intestine helps in breaking long chain polysaccharides to absorbable monosaccharides units. α-glucosidase is one of these enzymes that have gained considerable attention, targeting these enzymes would prevent the carbohydrate digestion and release of glucose in the blood stream (25). Most of the clinically used α-glucosidase inhibitors are acarbose, migitol and voglibose but due to several side effects of these drugs a less toxic-alpha glucosidase inhibitor is highly demanding (13).

Due to this, the antidiabetic potential of synthesized dihydropyrimidinone derivatives were evaluated using alpha-amylase and alpha-glucosidase assay. The *in silico* molecular docking and molecular dynamic simulation studies were also carried out to understand the binding mode of active ligand son the active binding site of protein. One of the tested compound **A** belonging to the class of DHMP showed positive results against antidiabetic activity by performing alpha glucosidase inhibition assay. The alpha-amylase inhibitory activity of these compounds did not show any potential inhibition by any of the ligands, however, in α-glucosidase activity, one of the compound (**A**) showed significant anti-diabetic activity. The compound **A** showed 81.99% inhibition of enzyme when compared to standard ascorbic acid that showed 81.18% inhibition. A very close inhibition of the enzyme by active ligand with the reference suggest potential role of this compound as selective a-glucosidase inhibitor. The IC_50_ of this compound determined by linear regression method showed to be 1.02 µg/ml. The structure activity analysis revealed that the compounds having alkyl substituents did not show any activity. Similarly, compounds having strong electron withdrawing groups (Compound **C** [-Cl] and Compound **D** [-F]) also did not show any inhibition of enzymes. Interestingly, the compound **A** having phenyl group with no substitution was effective against alpha-glucosidase activity. In a recent study, Bairagi and coworkers designed and synthesized a series of dihydropyrimidinone derivatives constituting electron withdrawing and electron donating groups on phenyl ring. The anti-hyperglycemic activity of those compounds were evaluated and their results revealed significant lowering of glucose level by the derivatives containing alkoxy group in the phenyl ring (12). In another study, *in vitro* anti-diabetic activity on α-amylase and α-glucosidase was conducted by Bharathi and coworkers against novel 10-chloro-4-(2-chlorophenyl)-12-phenyl-5,6-dihydropyrimido[4,5-a]acridin-2-amines. Their results also revealed significant inhibition of these enzymes by the synthesized derivatives (26). Karimian and co-workers also designed, synthesized and characterized a series of dihydropyrimidinone derivatives as β-glucuronidase inhibitor. Their study also revealed potent activity of one of these derivatives with IC_50_ of 31.52 ± 2.5 µM (27). Our results also revealed potential inhibitory activity of active compound against α-glucosidase.

In order to further explore the activity of active compounds against alpha-glucosidase the molecular docking analysis was performed. *In silico* tools are found to be very useful in the early prediction of pharmacokinetic and pharmacodynamic properties of new chemical entities as well finding novel targets for diseases through system biology approach (7, 28–30). The main purpose of the docking is to provide preliminary information about affinity of any compound before switching to *in vitro* experimentation. The docking analysis revealed stable protein ligand interaction having lowest binding energy score of -7.9 kcal/mol which was comparable to standard acarbose having lowest binding energy of -7.8 kcal/mol. The amino acid residues MET363 showed stable hydrogen bonding with the dihydropyrimidinone moiety. Some pi-alkyl and pi-pi stacked interactions were also observed with VAL867, LEU868, ARG608 and substituted benzene ring. The redocking analysis conformed the docking procedure by superimposing the already docked complex on the active binding site of redocked complex. The low rsmd of 0.1 kcal/mol revealed the docking procedure was validated. The molecular simulation studies were further performed to analyze the binding energies and the protein stability after complex formation. The study revealed the complex was stabilized at 100 s trajectories. The close rmsd of 1.7 A for the protein-ligand complex and the 1.6 A for the protein revealed the flexibility of the protein and stability of the complex. Moreover, the root mean square fluctuation also showed little fluctuation and stability of the both the complex and the protein. The consistent values of Rg were observed from the results and a good stability was observed due to similar behavior of compactness by the complex. The average SASA value for protein-ligand complex was 30906.17 nm2 (red), similarly, the average SASA value for protein complex was 30736.86 nm2 (blue). The values suggested no significant changes on the protein structure and the values were found to be relatively stable over the period of 100 ns. The calculations showed that the complex had a significant similar value of SASA with that of reference protein. The interaction energy for the protein-ligand complex at 100 ns was found to be -53.16 kcal/mol. The highest energy attained was -69.3 kcal/mol and showed favorable binding of ligands with hTERT. The results validated the molecular docking results and showed the potential of ligand as a-glucosidase inhibitor. The simulation results showed that the complex formed four hydrogen bonds concluding effective and tight bounding of ligands in the active biding site of α-glucosidase protein. The overall simulation data revealed the active ligand binds in the active binding site of protein with less fluctuation and lowest energy is required for the binding. The less difference in the energy, rmsd, rmsf and Rg revealed the protein does not fluctuates much while interacting with the ligand, suggesting it to be a good target for α-glucosidase.

## Conclusion

The present study revealed successful determination of anti-diabetic potential of *6-benzyl-4-(4-hydroxyphenyl)-3,4,6,7-tetrahydro-1H-pyrrolo[3,4-d]pyrimidine-2,5-dione* (Compound A) as α-glucosidase inhibitor. The inhibitory potential of this compound was shown to be 81% having IC_50_ of 1.02 µg/ml. The *in silico* molecular docking analysis is also consistent with the *in vitro* studies. The interaction pattern revealed important hydrogen bonding and electrostatic forces playing important role in binding. Moreover, the molecular dynamic simulation studies also showed stable interaction of ligand with the protein in the active binding site with less fluctuation and unfolding of protein during interaction. The energy required for interaction is also low suggesting the active compound has the potential to be developed as a selective new α-glucosidase inhibitor for the treatment of type 2 diabetes.

## Data availability statement

The datasets presented in this study can be found in online repositories. The names of the repository/repositories and accession number(s) can be found in the article/supplementary material

## Author contributions

UI, BN, RA: Project design and Concept, SM, MA, YD: Project Supervision, Project Administration, HZ, AP-S: Manuscript Proofreading and edition. All authors contributed in manuscript writing and edition. All authors contributed to the article and approved the submitted version.

## Funding

The funding was provided by ORIC, Riphah-ORIC-21-22/ FPS-51 and Riphah-ORIC-21-22/ FPS-56, Riphah International University.

## Acknowledgments

We would like to acknowledge ORIC, Riphah International University for funding this project.

## Conflict of interest

The authors declare that the research was conducted in the absence of any commercial or financial relationships that could be construed as a potential conflict of interest.

## Publisher’s note

All claims expressed in this article are solely those of the authors and do not necessarily represent those of their affiliated organizations, or those of the publisher, the editors and the reviewers. Any product that may be evaluated in this article, or claim that may be made by its manufacturer, is not guaranteed or endorsed by the publisher.
